# Targeting CREB in Cancer Therapy: A Key Candidate or One of Many? An Update

**DOI:** 10.3390/cancers12113166

**Published:** 2020-10-28

**Authors:** Luigi Sapio, Alessia Salzillo, Angela Ragone, Michela Illiano, Annamaria Spina, Silvio Naviglio

**Affiliations:** Department of Precision Medicine, University of Campania “Luigi Vanvitelli”, Via L. De Crecchio 7, 80138 Naples, Italy; luigi.sapio@unicampania.it (L.S.); alessia.salzillo@unicampania.it (A.S.); angela.ragone@unicampania.it (A.R.); michela.illiano@unicampania.it (M.I.); annamaria.spina@unicampania.it (A.S.)

**Keywords:** CREB, cancer therapy, drug inhibitors, GSKJ4

## Abstract

**Simple Summary:**

Only 5% of all drug-related targets currently move from preclinical to clinical in cancer, and just some of them achieve patient’s bedside. Among others, intratumor heterogeneity and preclinical cancer model limitations actually represent the main reasons for this failure. Cyclic-AMP response element-binding protein (CREB) has been defined as a proto-oncogene in different tumor types, being involved in maintenance and progression. Due to its relevance in tumor pathophysiology, many CREB inhibitor compounds have been developed and tested over the years. Herein, we examine the current state-of-the-art of both CREB and CREB inhibitors in cancer, retracing some of the most significant findings of the last years. While the scientific statement confers on CREB a proactive role in cancer, its therapeutic potential is still stuck at laboratory bench. Therefore, pursuing every concrete result to achieve CREB inhibition in clinical might give chance and future to cancer patients worldwide.

**Abstract:**

Intratumor heterogeneity (ITH) is considered the major disorienting factor in cancer treatment. As a result of stochastic genetic and epigenetic alterations, the appearance of a branched evolutionary shape confers tumor plasticity, causing relapse and unfavorable clinical prognosis. The growing evidence in cancer discovery presents to us “the great paradox” consisting of countless potential targets constantly discovered and a small number of candidates being effective in human patients. Among these, cyclic-AMP response element-binding protein (CREB) has been proposed as proto-oncogene supporting tumor initiation, progression and metastasis. Overexpression and hyperactivation of CREB are frequently observed in cancer, whereas genetic and pharmacological CREB downregulation affects proliferation and apoptosis. Notably, the present review is designed to investigate the feasibility of targeting CREB in cancer therapy. In particular, starting with the latest CREB evidence in cancer pathophysiology, we evaluate the advancement state of CREB inhibitor design, including the histone lysine demethylases JMJD3/UTX inhibitor GSKJ4 that we newly identified as a promising CREB modulator in leukemia cells. Moreover, an accurate analysis of strengths and weaknesses is also conducted to figure out whether CREB can actually represent a therapeutic candidate or just one of the innumerable preclinical cancer targets.

## 1. Introduction

GLOBOCAN 2018 has confirmed for the oncological disorders the dubious distinction of being the second leading cause of death worldwide [1]. Despite the efforts made in drug discovery over time, the achieved results have not always met recovery and survival expectation [2]. Indeed, although shocking numerous therapeutic targets are constantly recognized in cancer models, only 5% of drug-related targets generally reach the clinical trials, and a smaller portion receives the Food and Drug Administration (FDA) approval for cancer [3]. Moreover, clinical effectiveness of those drugs is sometimes related to off-target rather than their putative targets [4]. What reasons could explain this discrepancy? The inability to predict the patient responses before moving to clinical trials could represent a valid but partial explanation regarding the emerging “big paradox”. Therefore, scientists have increasingly questioned themselves about this, identifying intratumor heterogeneity (ITH) and preclinical cancer models as a source of the current frustrating drug discovery failure [5,6]. Assumed for the first time by Julian Huxley in 1958, ITH has become factual with the advent of next-generation sequencing [7]. ITH is currently defined an evolutionary framework in which spatially and temporally distinct genomic alterations affect subsets of cancer cells, generating a branched shape within the tumor [8]. Unlike what was improperly claimed before, ITH involves not only coding sequences but also epigenetic regulatory mechanisms [9]. Even though ITH characterization has just begun, and more comprehensive studies are needed to fully recapitulate the cancer genome evolution, its engagement in drug resistance and tumor mortality is broadly accepted [10]. Even though Seth and colleagues recently demonstrated a similar clonal hierarchy even in patient-derived xenograft (PDX) pancreatic cancer models, a reduced ITH of existing preclinical models is considered a causative element for targets failure in clinical [11,12]. With the purpose of increasing ITH index and recapitulating the human disease features, novel and innovative preclinical cancer tools have been developed over the years, such as patient-derived organoids [13]. Significantly, the in-vivo perspective is more complex, where a wide variety of murine tumor models makes any consideration complicated. Recently, Guerin et al. critically examined the advantages and weaknesses of the most employed preclinical mouse models in drug discover design [14]. Unfortunately, collective interactions among tumor, immune and stroma cells remain challenging to replicate in preclinical models, as well as their relative influence on ITH. In light of these remarks, no existing preclinical model can perfectly forecast the patient outcomes in cancer. Therefore, only a combined multidisciplinary approach, aimed prior to examine the relevance of preclinical findings, could reduce phase II and III failure in cancer. In addition, verifying their relative targets and having a deep knowledge of the molecular mechanism of such anticancer drugs would help to propose more efficient therapy options in clinical [15]. Considering the above, the present review is conceived to investigate the feasibility of targeting CREB in cancer treatment. Starting with an essential but comprehensive overview on CREB cellular functions and pathways, we then focus on the existing evidence regarding the CREB engagement in cancer pathophysiology, from the oldest to the most recent. Furthermore, the CREB inhibitor state-of-the-art is investigated extensively, including the histone lysine demethylases JMJD3/UTX inhibitor GSKJ4 that we newly identified as a promising CREB modulator in AML cells [16]. In conclusion, analyzing the strengths and weaknesses of targeting CREB, we attempt to figure out whether this therapeutic approach can actually represent a viable solution in cancer in the next future.

## 2. Functions and Signaling Pathways of CREB Transcription Factor

Identified for the first time in 1987, CREB is a member of basic leucine zipper (bZIP) transcription factors, which also include c-Myc, c-Fos and c-Jun [17]. Beyond the proper CREB, two additional closely related DNA-binding proteins, namely cAMP response element modulator (CREM) and activating transcription factor (ATF), recognize what is referred to as the CREB superfamily [18].

CREB1 gene is located on the long arm of chromosome 2 and encodes for three distinct CREB isoforms [19]. Despite their identical cell function and expression, different tissue-related isoenzymatic profiles were detected [20]. Recently, additional mRNA splice variants have been discovered in testis, where the loss of function and the identification of functional-nonfunctional CREB dimer has speculated the existence of supplementary regulatory mechanisms [21].

CREB1 is translated into a protein of 341 amino acids in length and 36,688 kDa in size, which recognizes the palindromic cAMP response elements (CRE) sequence binding both the full-length 5′-TGACGTCA-3′ and the preserved half string 5′-TGACG-3′ [19]. Schematically, four functional domains have been identified and termed, from N-terminus to C-terminus, as follows: (i) basal transcriptional activity domain, which cooperates with TATA-binding protein, endorsing the related genes transcription [22]; (ii) kinase inducible domain (KID); (iii) glutamine-rich domain, required for constitutive CREB activation and to modulate both residence time and transcriptional activity [23]; and (iv) bZIP domain, which allows CREB dimerization and increases CRE binding affinity [24]. Among others, KID domain surely denotes an essential core in controlling CREB activation. Indeed, critical pathway effectors induce selective post-translational modifications (PTMs) in KID residues, affecting its interaction with KIX domain of both coactivators, CREB-binding protein (CBP) and p300 [25,26]. Serine KID residues, for instance, are involved in signal-mediated CREB phosphorylation, and among them serine 133 (Ser133) definitely represents the most extensively studied KID-related PTM [27]. Phosphorylated by different protein kinases, such as protein kinase A (PKA), protein kinase B (PKB/AKT) and extracellular signal-regulated kinase 1/2 (ERK1/2), Ser133 primarily mediates CREB dimerization allowing the mutual recognition of CBP and p300 [28,29]. Additionally, CREB Ser133 residue also constitutes the ultimate target for depolarization-activated Ca(2+)-calmodulin-dependent protein kinases (CaM kinases) I and II [30]. Apart from the homodimeric form, CREB can be combined with other bZIP transcription factor members, such as c-Jun, CREM and ATF, generating heterodimer shape [31]. Besides CREB activation, other Ser133-related features have been proved, specifically related to CREB stability and degradation [32,33]. Nevertheless, its significance in CREB activation was strongly questioned by Briand and colleagues, who recently demonstrated that Ser133 phosphorylation is not required for CRE binding in learning and memory in-vivo models [34]. Accordingly, different studies designate Ser133 CREB phosphorylation essential but not sufficient to ensure the CRE-related gene transcription, identifying additional coactivators in the gene expression control, such as CREB-regulated transcription coactivators family (CRTCs) [35,36]. Despite contradictory findings, changes in Ser133 phosphorylation status, as well as in other KID-related serine residues, are still considered an explicit signal to govern CREB transcriptional activity. In this regard, dissimilar and sometimes opposite consequences have been associated with KID serine residues phosphorylation. Indeed, while phosphorylation of Ser129 and Ser133 positively affects CREB transcription activity, the same PTM in Ser111 and Ser121 completely blocks the CREB-related genes expression [37].

Considering the huge number of regulated genes, several cellular mechanisms are strictly dependent on CREB activation, and consequently on its phosphorylation status. Inflammation, DNA repair, immune response and cell cycle progression are just some of the cellular processes in which CREB engagement has been confirmed [38]. The existence of other CREB-related enzymatic modifications, such as ubiquitination, methylation, glycosylation and SUMOylation, further support the relevance of controlling CREB in cellular homeostasis [39,40,41]. In addition to PTMs, additional protein-unrelated regulations impact on CREB activity. Recent studies have described how small non-coding RNAs control CREB expression in cancer and cerebral ischemic injury [42,43].

Considered one the leading factors in nervous system development, CREB affects synaptic plasticity, memory, neurotransmission and neuronal survival [44]. Therefore, CREB dysregulations are largely associated with the onset of neuropathological and neurodegenerative diseases, including schizophrenia and Alzheimer’s disease (AD) [45,46].

In this connection, characterizing the N-[2-(3, 4-dimethoxyphenyl) ethyl]-3-phenyl-acrylamide (gx-50) compound as a novel neuroprotective agent, Tang and collaborators further proved the involvement of glycogen synthase kinase-3 (GSK-3)/CREB pathway in AD. Indeed, in-vivo results showed that Aβ-treated cerebral cortex neurons exhibited a significant reduction in both GSK-3 and phospho-Ser133 CREB, whereas gx-50 pre-treatment prevented the Aβ-induced GSK-3 and CREB downregulation [47]. While the reduced CREB activation is considered a hallmark of neurological disorders, overexpression and hyperphosphorylation are frequently associated with neoplastic diseases. This latter aspect, together with the characterization of CREB involvement in cancer, is extensively debated in the following section.

## 3. Recent Advances in Tumor Pathophysiology: The Relevance of CREB Engagement

**Figure 1:**
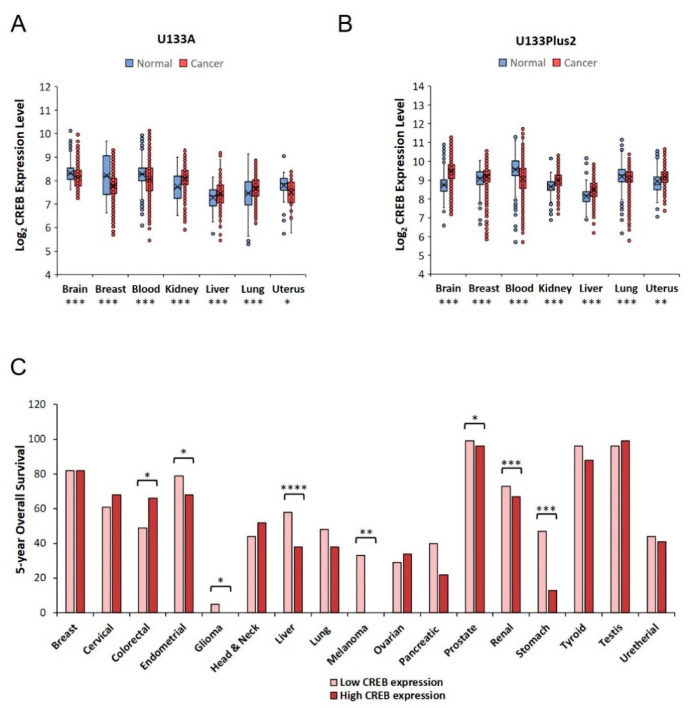
CREB expression levels in healthy and cancer patients. Gene Expression database of Normal and Tumor tissues 2 (GENT2) open access database (http://gent2.appex.kr) was employed to evaluate CREB expression levels in cancer and non-cancer tissues within Affymetrix U133A (**A**) and U133Plus2 (**B**) platforms. The significantly shared groups between these two distinct microarrays are plotted as tissue boxplots. (**C**) Based on CREB mRNA content, Kaplan–Meier plots of seventeen types of tumor were analyzed using the interactive open-access Human Protein Atlas (HPA) database (www.proteinatlas.org/pathology). For each analyzed cancer type, median five-year overall survival values of both stratified groups are plotted as columns in the histogram graph. * *p* < 0.05, ** *p* < 0.01, *** *p* < 0.001, **** *p* < 0.0001 by two-sample *t*-test.

Among the over 4000 CREB-regulated genes, a conspicuous number is actively involved in uncontrolled cell proliferation and tumorigenesis, thus suggesting a pivotal CREB participation in several cancer-related mechanisms. What might just seem a commutative concept is actually supported by multiple lines of evidence in which the role of CREB in cancer pathophysiology has markedly been proved [19]. Several studies have formerly recognized CREB as a putative oncogenic signaling in different tumor types, especially in leukemia and glioma [48,49]. The advent of high-throughput technologies has further contributed to enrich the knowledge base about CREB engagement, demonstrating a high susceptibility of this gene to be dysregulated in cancer. Using the latest Gene Expression database of Normal and Tumor tissues 2 (GENT2) tool, we analyzed the CREB expression levels in more than 60,000 human samples stratified into tumor and non-tumor (normal) subgroups [50]. GENT2 collects gene expression data from two distinct microarray platforms (Affymetrix U133A and U133Plus2) containing 44,000 and 23,000 samples, respectively. Querying GENT2 for CREB expression pattern, significant alterations have been recorded between cancer and non-cancer samples. Specifically, we observed CREB alterations in 13 of the 31 paired tissues contained within U133A, whereas U133Plus2 results in 21 out of 37 groups in which CREB appeared dysregulated. Comparing these two significant clusters, seven distinct tumors featured in both microarrays, namely blood, brain, breast, kidney, liver, lung and uterus (Figure 1A,B).

Despite the loss of cancer specificity, combined RNA-seq data from The Cancer Genome Atlas (TCGA) and Human Protein Atlas (HPA) platforms reveal a negative correlation between CREB expression levels and overall survival (OS) in specific tumor subtypes, speculating its potential role as a prognostic factor [51]. Figure 1C displays the clinical outcome of about 8000 patients who, classified by tumor types, have been further separated into two subcategories (Low and High expression levels) based on CREB mRNA content. While the obtained results disclose a worse CREB-related prognosis in several types of cancer, such as endometrial, glioma, liver, melanoma, prostate, renal and stomach, an inverse correlation comes to light exclusively for colorectal cancer where it seems that a high CREB expression improves the related OS (Figure 1C).

With the purpose of characterizing the CREB engagement in cancer pathophysiology, in this section, we report some of the major recent advances. However, as a consequence of both outspread of available data and limited manuscript length, this approach is applied only for certain tumor types.

### 3.1. CREB and Melanoma

Melanogenesis represents a critical cellular process involved in melanocytes differentiation, proliferation and hyperpigmentation [52]. It is generally upregulated in skin-related tumors where it promotes the malignant phenotype supporting uncontrolled cell growth [53]. Therefore, it is finely tuned by both extracellular and intracellular signals, including CREB [54]. For instance, among the pigmentation-related hormones, alpha melanocyte stimulating hormone (α-MSH) binds melanocortin-1 receptor (Mc1r) stimulating adenylate cyclase activity and triggers cAMP/PKA/CREB pathway [55]. Additionally, CREB negatively regulates both activating protein-2 (AP-2) and cellular communication network factor 1 (CCN1/CYR61), promoting cell growth and tumor angiogenesis in A375SM and C8161-c9 high metastatic melanoma cells [56,57].

More recently, an extensive interaction between microphthalmia-associated transcription factor (MITF) and CREB has also been proved [58]. Considered as one of the leading melanogenesis regulators, MITF is generally amplified in roughly 20% of melanoma patients [59]. Interestingly, recent evidence demonstrates the existence of a cAMP–CREB–MITF axis wherein a high CREB expression may induce a subsequent activation of MITF, upregulating genes engaged in melanin biosynthesis, such as TRP-1 and TRP-2 [60,61]. Phenotypically, MITF downregulation via CREB signaling has been reported to provoke melanogenesis blockage affecting cell viability and body pigmentation in B16F10 murine melanoma cells and zebrafish model, respectively [62,63]. Despite the relevance in melanoma progression, MITF is currently classified as a not-druggable target; therefore, modulating its upstream pathways, including CREB, could represent an alternative viable approach to control MITF activity in melanoma. In this regard, different studies have shown how pharmacological inhibition of CREB, as well as CRTC1 and CRTC3 coactivators, leads to anti-melanogenic effects abrogating MITF expression in both melanocyte cells and ex-vivo human skin cultures [64,65]. To the same end, perturbation of the CREB upstream kinases may also facilitate the MITF-mediated melanogenesis shutdown and thus melanoma progression [66]. Involved in MITF regulation, the essential Wnt/β-catenin signaling pathway can also cooperate with CREB, either directly or indirectly [67,68].

Besides MITF modulation, CREB can negatively control the expression of adenosine deaminase acting on RNA 1 (ADAR1) enzyme, which is implicated in A-to-I RNA editing of mRNAs and microRNAs [69]. Shoshan and coworkers proved an inverse correlation between phospho-CREB and ADAR1 levels in both low and high metastatic melanoma cell lines [70].

CREB-related metabolic features have also been described in non-coetaneous melanoma. Consistently, Voropaev’s group provided evidence that CREB loss of function drastically decreases both in-vitro and in-vivo UVEAL melanoma cells progression, mainly via GLUT-1 repression [71].

### 3.2. CREB and Gastric Cancer

Several studies have emphasized the high frequency of CREB to be dysregulated in gastric cancer (GC) over the years [72]. Indeed, analyzing CREB expression in more than 200 gastric samples, including non-tumor and both primary and metastatic tumors tissues, Wang and collaborators suggested an intriguing linkage between CREB levels and metastasis, tumor stage and clinical outcome [73]. Beyond the clinical evidence, different molecular mechanisms have been described to be involved in CREB-mediated oncogenic effects in GC. Firstly, CREB has been proved to operate as an upstream effector of the CCAAT/enhancer-binding protein beta (C/EBPβ), a transcription factor frequently overexpressed in GC and associated with the suppression of the differentiation marker TFF1 [74]. Moreover, p38-mediated CREB activation can also repress the pH regulator carbonic anhydrase IX (CA9) expression [75]. In detail, the authors described a regulation model wherein p300 serves as a link between CREB and SIRT1, which effectively regulates CA9 expression. Intriguingly, while CA9 is generally overexpressed in a large number of solid tumors, it is downregulated or totally absent in GC [76]. The close connection between CREB and SIRT1 was also investigated by Zhang et al. who, studying miR-132 in Lgr5+ stem GC cells, showed that SIRT1 deacetylates CREB and, suppressing its phosphorylation, downregulates the expression of the CREB-related genes [77].

Supporting the CREB relevance in GC, CREB knockdown experiments inhibited cell viability and colony formation in BGC-823 and SGC-790 GC cells, inducing G0/G1 phase arrest and repressing the expression of its downstream targets, such as cyclin D1, BCL2 and MMP-9 [78].

Furthermore, CREB activation has been associated with chemotherapy resistance in GC. Specifically, Zhang and colleagues pointed at CREB Ser-133 as one of the causes of the cisplatin resistance in Lgr5+ cells [77]. Similarly, Li et al. displayed that upregulation of mitochondrial ribosomal protein L33 (MRPL33)-short (S) promotes epirubicin responses, deactivating the PI3K/AKT/CREB axis [79].

### 3.3. CREB and Leukemia

As is widely known, leukemogenesis is deeply regulated by CREB signaling. During this process, specific hematopoietic growth factors such as GM-CSF and IL-3 stimulate CREB activation and promote both proliferation and survival of myeloid and lymphoid progenitor cells [80]. Its physiological distinction makes CREB a decisive factor for the blood-related malignant transformation as well. In this connection, CREB overexpression has been proposed to support uncontrolled cell growth and apoptosis repression in hematopoietic-derived lineage, stimulating the survival-related genes expression, such as Bcl-2, Mcl-1, Bcl-xL, survivin and XIAP [48,81]. Additionally, in different preclinical leukemic models, CREB activation leads to an inappropriate cyclin A1 and D2 expression with the purpose of pushing cell cycle progression and growth [82,83].

More recently, novel CREB axes have been described in AML [84,85]. In detail, in zebrafish AML models, CREB overexpression increases CCAAT-enhancer-binding protein-δ (C/EBPδ) provoking myeloid differentiation blockage and triggering monocytic leukemia [84]. Furthermore, exploring nuclear scaffold lncRNA MALAT1 therapeutic candidacy in CML, Balasis and coworkers tested the consequences of MALAT1 antisense oligonucleotides (ASOs) and all-trans retinoic acid (ATRA) combination treatment in CMML patient-derived xenografts. Alongside the reduced cells engraftment in the presence of both compounds compared to ASO and ATRA single treatment, they first identified CREB as MALAT1 target genes [85]. In this connection, Pigazzi and colleagues identified miR-34b as one the main non-coding RNAs implicated in CREB modulation in AML [86,87]. Indeed, they initially demonstrated that miR-34b is generally downregulated in AML patients, inducing CREB overexpression and promoting leukemia cell proliferation [86]. Subsequently, with the purpose of explaining miR-34b depletion in myeloid malignancies, the authors also examined the methylation status of its promoter in patient-derived AML cells, observing a recurrent hypermethylation in 66% of the analyzed cases [42].

While CREB overexpression promotes and sustains leukemogenesis, on the contrary, its downregulation is generally associated with diminished leukemic burden. Indeed, it has been reported that CREB knockdown reverts tumor state in several leukemia cell lines [81,87]. Shabestari and colleagues demonstrated that loss of CREB expression triggers caspase-mediated apoptosis and reduces pro-survival signaling in pre-B acute lymphoblastic leukemia cells [88]. Similarly, additional studies demonstrate that CREB repression via miR-22 overexpression drastically impairs proliferation index in THP-1, KOCL-48 and MV4-11 cells, both in-vitro and in-vivo [89].

### 3.4. CREB and Brain Cancer

Beyond having a relevant role in embryonic brain development, CREB-mediated transcriptional activity is required for gliomas regulation and maintenance [90,91]. Usually, CREB levels correlate with glioma tumor grades, especially with regard to grade III and IV wherein a high expression has been observed [92].

Gliomas include tumors with different genetic signatures, chemotherapy responses and clinical patters. From this perspective, Barresi et al. tried to discriminate the two major glioma subtypes based on phospho-CREB expression [93]. Interestingly, the entirety of the analyzed astrocytic tumors expressed p-CREB, whereas only 46% of oligodendrogliomas showed appreciable activation levels. Mechanistically, MAPK and PI3K mediated CREB activation has been detected in both neural stem progenitor cells (NSPCs) and glioblastoma cells [94]. More recently, mutant and loss of function experiments have further supported the relevance of PI3K/CREB axis in this cancer type. Indeed, if on the one hand PIK3CAH1047A oncogenic mutant expression spontaneously generated brain tumors in GEMM models, on the other hand PTEN abrogation intensely reduced the high-grade glioma invasiveness [95]. Besides the canonical cAMP/PKA pathway, CREB activation can also be triggered by CDK5 [96]. Specifically, Mukherjee’s group established that CDK5 pharmacological inhibition restrains glioma stem cell renewal in xenografted drosophila models, partially by reducing PKA-independent CREB activation. Additionally, binding the promoter region, CREB is also found to be partially responsible of the PGC1α regulation, modulating the metabolic reprogramming of GBM cells in normal astrocyte cells [97].

As a transcription factor, CREB can be either target for specific miRNAs or modulator for miRNAs expression. By using the luciferase reporter system and real time PCR, Geng and coworkers identified CREB and KLF as a functional upstream regulator of the tumor suppressor miR-132 in U87 and U251 astrocytoma cells [98]. On the contrary, CREB abrogation by miR-1224-5p and miR-200b suppresses glioma progression in preclinical models [99,100].

### 3.5. CREB and Testis

A close correlation between intracellular CREB levels and normal spermatogenesis has been discussed extensively over the years [101,102]. For instance, Sertoli and Leydig cell metabolism is regulated by pituitary gonadotropins FSH and LH, which, in turn, activate CREB via adenylate cyclase promoting the transcription of essential genes for germinal cell differentiation [103,104]. Despite the increasing number of findings revealing CREB significance in spermatogenesis, very little evidence reports its engagement in testicular carcinoma (TC). In this respect, CREB nuclear reduction has been proved to suppress gonadotropin-mediated steroid secretion through TNF abrogation in MA-10 Leydig cancer cells [105]. In addition, rolipram-induced CREB activation has been reported to ameliorate the acute irradiation damage on testicular dysfunction [106]. Moreover, different datasets show a reduction in CREB expression levels in TC tissue compared to the normal one [50,107]. The clinical and pathological significance of this interesting information remains largely unexplored.

## 4. How Far Is the Design of CREB Inhibitor in Clinical?

**Figure 2:**
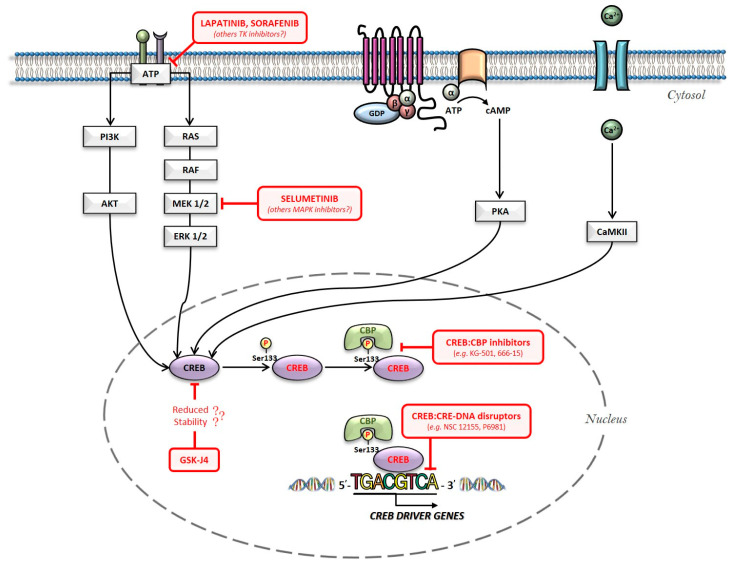
Overview of the main intracellular pathways involved in CREB activation: possible pharmacological hubs for its inhibition.

Taking into consideration CREB involvement in neoplastic transformation, CREB blockage is increasingly becoming a potential therapeutic strategy for cancer care [19]. In this respect, we previously described that adiponectin exposure strongly prevents Ser133-CREB phosphorylation in A549 non-small cell lung cancer (NSCLC), affecting both cell proliferation and division [108]. Additionally, we also observed aberrant CREB activation in six different NSCLC tissues compared to contiguous normal one, speculating CREB as a viable therapeutic strategy in this cancer type. We first reported the ability of the JMJD3/UTX demethylase inhibitor GSK-J4 of modulating CREB protein levels in AML cells, one of the leading CREB-dependent tumors [16]. Besides our findings, several CREB modulators have been developed and investigated as chemical probes in cancer diseases over the years. The current pharmacological and chemical approaches are based on two distinct design strategies that can be schematically termed as “CREB inhibitors” and “CREB-related pathways inhibitors” (Figure 2) [37]. Comprehensively, “CREB inhibitors” include small chemical compounds that selectively hamper CREB transcriptional activity, restraining either CREB:CBP or CREB:CRE-DNA interaction. Instead, “CREB-related pathways inhibitors” comprise molecules that regulate intermediates of distinct signaling pathways, which include CREB as final effector. Even though the latter approach does not directly affect CREB, emerging studies have highlighted the capability of this strategy of achieving appreciable results in terms of CREB inhibition, representing a potentially faster scenario for clinical applications [109,110].

For each examined compound, the planning and development evidence is summarized and reported below (Table 1). Potential critical issues are marginally handled in this subsection, whereas a more analytical reasoning is debated in the last part of the present review.

### 4.1. CREB:CBP Inhibitors

Renowned as KG-501, 2-naphthol-AS-E-phosphate represents the first moderately potent CREB inhibitor capable of disrupting CREB:CBP interaction [111]. Using a preexisting library, containing more than 760 compounds employed for ligand uncovering and binding site identification, Best and coworkers identified KG-501 as a CREB:CBP blocker using an NMR-based approach. In detail, they demonstrated that KG-501 disrupts CREB(KID):CBP(KIX) interaction by directly targeting phospho(Ser-133) CREB with a Ki of ~90 μM. However, it should be kept in mind that, even though KG-501 has been identified as CREB:CBP disruptor, other KIX-dependent transcription factors have been reported to be equally modulated by this compound, such as NF-κB and the proto-oncogene Myb. In this regard, it has been demonstrated that KG-501 prevents IL-1β mediated angiogenic stimulation, inhibiting both NF-κB and CREB transcriptional activity in NSCLC cell lines [112]. In addition, Uttarkar and colleagues recently recognized in leukemia cells a greater KG-501 mediated Myb–KIX inhibition comparted to CREB–KIX disruption [113]. Therefore, it is conceivable to assume KG-501 is a KIX domain disruptor, rather than a selective KID:KIX splicer. However, different mechanistic and chemical features remain partially unclear, including the linkage between other KIX-related transcription factors and CREB impairment. Despite its reduced specificity in CREB inhibition, anti-tumor mediated KG-501 properties have been reported in different preclinical models over the years [84,114,115].

To reach an optimized KID:KIX inhibitor, several molecules sharing a similar naphthol AS-E-phosphate structure have been successively developed and tested. However, most of them showed a very low solubility in water and unsatisfactory results in terms of binding and transactivation by NMR [111]. An analogous approach was also employed by Lee’s group, who identified, using the KG-501 structure as a query, Naphthol AS-TR-phosphate as the most powerful compound [116]. Nearly simultaneously, Park and coworkers further confirmed the performing properties of naphthol-AS-TR-phosphate, especially in terms of antiproliferative effects in different in-vitro and in-vivo lung cancer models, even though, performing microarray analysis of the naphthol-AS-TR-phosphate treated samples, they observed a dramatic E2F8 downregulation besides CREB-related pathway involvement [117]. Since no supplementary information is available concerning the mechanisms by which this compound regulates E2F8 expression and how naphthol-AS-TR-phosphate induces anti-tumor effects in lung preclinical cancer models, these findings leave a lot of unanswered questions about the ability of this molecule to inhibit the KID:KIX interplay selectively.

Investigating the scientific literature for other naphthol-derived compounds, the dephosphorylation product of the naphthol AS-E-phosphate has been reported to be more active in disrupting KID:KIX interaction compared to phosphate-containing compound [118]. Consistent with these findings, subsequent studies, aimed at analyzing the naphthol AS-E-phosphate conformation in tissue culture media, recognized the dephosphorylated form as the most representative one [119]. In view of all this, we might assume a hypothetical model in which removing the phosphate group represents a key factor for the biological activity of the naphthol AS-E-phosphate. Nevertheless, no data sustain this assumption also because the potential identification of cellular enzymes capable of converting KG-501 into dephosphorylated form is currently lacking.

The reduced permeability and biological activity represent the main limitations of all naphthol-derived compounds; therefore, to overcome these deficiencies, a new KID:KIX disruptor named 666-15 was discovered in 2015 [120]. However, based on the existing findings, the involvement of other independent KID:KIX mechanisms in the 666-15 mediated CREB blockage, as well as the ability of this compound of inhibiting other transcription factors, cannot be ruled out completely. 666-15 strongly inhibits cell proliferation in different cancer models at very low micromolar concentration without affecting normal cell proliferation [121,122,123]. In addition to reducing tumor progression, in pancreatic ductal adenocarcinoma (PDAC), the 666-15 compound also impacts on the intratumor microenvironment, increasing immune infiltration of both CD3+ and CD8/CD4 T cell ratio [124]. Although non-cancer beneficial therapeutic effects have also been described for 666-15, such as prevention of ROS-induced cardiac hypertrophy, serious cellular damage has been reported specifically against central nervous system [124,125,126]. In the effort of improving both solubility and bioavailability, a novel ester prodrug of 666-15 was recently identified by Xie and coworkers [127].

### 4.2. CREB:CRE-DNA Inhibitors

Arylstibonic acids currently represent the most relevant CREB:CRE-DNA class of inhibitors [37]. Identified by high-throughput fluorescence–anisotropy screening of about 2000 compounds, arylstibonic acids have been selected thanks to their ability of disrupting CREB b-ZIP binding to DNA [128]. Among others, NSC 12155, NSC 13,778 and NSC 45,576 have shown a very higher specificity in inhibiting CREB:CRE-DNA complex and, therefore, they are discussed hereafter. Commercially known as surfen, NSC 12,155 affects NIH3T3 and HER-2/neu+ cell proliferation directly modulating CREB transcriptional activity [129]. Besides the CREB-related effects, other surfen-mediated consequences have been reported in tumor and non-tumor systems, identifying it as an anti-invasiveness, anti-inflammatory and anti-remyelination agent [130,131].

The current evidence proposes NSC 13,778 (stibavirin) as a widespread b-ZIP blocker rather than a specific CREB b-ZIP inhibitor. Indeed, comparative studies have highlighted the ability of this compound to inhibit Fos/JunD, C/EBPβ and VBP [128,132]. In addition, stibavirin impedes the interaction between TFE3 transcription factors and their respective promoters [133]. Despite the lack of cancer-related evidence, other pharmacological applications have been attested for NSC 13778. Notably, binding to CD4+ T cells, stibavirin has been described as HIV-1 entry inhibitors [134]. Finally, P6981 compound is an NSC 13,778 derivative which better suppresses DNA binding of CREB in respect with stibavirin [135].

Even though NSC 45,576 has been recognized as CREB-CRE disruptor, it has also been shown to be a potent Exchange Proteins directly Activated by cAMP (EPAC) inhibitor, without affecting PKA activity [128,136]. Due to commercial unavailability and difficult to prepare, NSC 45,576 has not been yet tested in preclinical cancer studies.

### 4.3. CREB-Related Pathways Inhibitors

Suppression of signaling pathway intermediates that physically phosphorylate CREB could depict an alternative pharmacological strategy in order to modulate CREB therapeutically. In this regard, aberrant activation of CREB upstream kinases, such as PKA, PKB/AKT and mitogen-activated protein kinases (MAPK), represents one of the main causes of CREB hyperactivation in cancer [137].

Considering the massive numbers of related studies, and the limited space available for this review, we decided to apply stringent searching criteria. In detail, we firstly looked for CREB upstream kinases inhibitors currently under investigation by clinical trials in cancer, with the purpose of providing the closest experimental treatment for approval. Thereafter, we enclosed for all compounds that expressively impaired CREB function, and finally we further screened them for cancer types, selecting those in which CREB was significantly overexpressed and/or associated with a worse OS.

Even though CREB is considered the main downstream of PKA signaling, no clinical trials are currently ongoing regarding this pathway. Indeed, only preclinical evidence has been published thus far, confirming the ability of these compounds of modulating CREB preferentially [138]. Concerning the AKT pathway, even though several clinical trials are testing the efficacy of different AKT modulators, such as MK-2206, GDC-0068 and AZD5363, their effects on CREB-mediated transcriptional activity remain unclear [139]. Conversely, emerging evidence recently emphasizes how some tyrosine kinase inhibitors (TKIs), such as lapatinib and sorafenib, exert both antiproliferative and proapoptotic properties directly impacting on CREB activation [109,110,140,141].

As a selective dual epidermal growth factor receptor (EGFR) and the human epidermal growth factor receptor 2 (HER-2) inhibitor, lapatinib (GW572016) was approved for metastatic and trastuzumab-resistance HER2-positive breast cancer treatment by FDA in 2007 [142]. Mechanistically, it inhibits the downstream EGFR and HER2 signaling pathways, such as MAPK, PI3K-AKT and PLCγ, even though lapatinib-mediated CREB modulation has also been reported in SKBR3 and T47D breast cancer cell lines [110,140]. On that note, a recent randomize phase III study (TyTAN), enrolling 261 patients affected by advanced gastric cancer, revealed that combination therapy with lapatinib plus paclitaxel significantly increases median OS, progression-free survival (PFS) and overall response rate (ORR) compared to paclitaxel alone (OS: 11 vs. 8.9 months; PFS: 5.4 vs. 4.4 months; and ORR: 27% vs. 9%) [143].

Sorafenib (BAY-43-9006) is a multi-target kinase inhibitor approved as the first-line therapy for advanced renal cell carcinoma and unresectable hepatocellular carcinoma [144]. Studying the combinatory effects of sorafenib and red ginseng extract, Kim and colleagues recently exhibited a partial attenuation in CREB phosphorylation made by sorafenib treatment in renal cell carcinoma [141]. Consistently, analog results have also been reported by independent research [109]. Different clinical studies have proved the safety of sorafenib plus chemotherapy agents in several malignances [145,146]. Concerning the efficacy, ECOG 5203 trial (*n* = 44) reports how the sorafenib–docetaxel–cisplatin cocktail therapy has shown promising results in patients with inoperable metastatic or locally advanced gastric or gastroesophageal cancer [147].

Although several MAPK or PI3K inhibitors are already in clinical or in trials, their specific outcome on CREB activation has been poorly documented and only for some, such as selumetinib (AZD6244 and ARRY-142886) [148]. Approved for the treatment of pediatric patients with neurofibromatosis type-1 and inoperable plexiform neurofibromas, selumetinib is a selective non-ATP-competitive small-molecule inhibitor of mitogen-activated protein kinase 1 and 2 (MEK1/2) which directly affects ERK1/2 activation [149]. In randomized phase II clinical trial, involving 385 patients affected by advanced cutaneous or unknown primary melanoma, the combination selumetinib plus dacarbazine showed a significant advantage in PFS compared with single treatment group (PFS: 5.6 vs. 3.0 months) [150].

## 5. GSKJ4 as a Novel CREB Inhibitor in AML Models

H3K27 methylation (H3K27me) status has a crucial impact on the expression of several genes actively involved in cell differentiation and proliferation, and thus it is finely regulated by two opposite enzyme classes which promote methylation and demethylation, respectively [151]. Increasing evidence ascribes to these histone modifiers the leading causes of the H3K27me dysregulation in precancerous and cancers lesions [152,153,154]. Therefore, targeting of H3K27 methylation-modulating enzymes has posed as a potential therapeutic approach in cancer therapy [155]. Derived from the pioneer GSK-J1, GSK-J4 is a cell-permeable UTX and JMJD3 blocker capable of affecting cell growth and survival especially in glioma and leukemia cells, where the H3K27me dysregulation occurs recurrently [156,157,158]. Additional GSK-J4 mediated antiproliferative effects have also been reported in other tumor types, such as breast, lung and prostate cancer cells [159,160,161]. According to these findings, we recently demonstrated that forskolin increases leukemia cell-sensitivity to GSK-J4 through apoptotic cell death induction and cAMP/PKA/CREB involvement [162]. Starting from these results, we observed that GSK-J4 dramatically downregulates CREB protein in leukemia cells, proposing the UTX and JMJD3 inhibitor as a potential newly CREB modulator [16].

In detail, we reported that GSK-J4 treatment significantly decreases CREB protein level in three different AML cell lines without affecting CREB mRNA expression levels. To support the hypothesis that no transcriptional regulations are involved in the GSK-J4 mediated CREB downregulation, experiments aimed at investigating the consequences of GSK-J4 on microRNA-34b, the most relevant small non-coding CREB RNA in leukemia, were also performed. Surprisingly, GSK-J4 further reduced miRNA-34b expression, excluding this specific CREB regulation mechanism as a possible explanation for the GSK-J4 mediated CREB modulation. Simultaneously, with the purpose of evaluating CREB half-life and proteasome-engagement in response to GSK-J4 exposure, cycloheximide and MG 132 were specifically employed. Experimental results indicated that CREB protein stability dramatically drops in reaction to GSK-J4 administration, whereas proteasome impairment mostly hinders the GSK-J4 induced CREB downregulation. Additionally, we provided comprehensive features regarding the related mechanisms of action, because we also reported a rapid PKA-mediated CREB phosphorylation that clearly predates CREB degradation as a function of GSK-J4 impact on AML cells. The PKA involvement was finally corroborated by H89 compound, which, inhibiting the cyclic AMP-dependent kinase, nearly totally impeded both the GSK-J4 mediated CREB phosphorylation and downregulation.

## 6. Strengths and Weaknesses of Targeting CREB in Cancer

Despite the recent advances, what we schematically term in this review as “CREB inhibitors” are only employed in preclinical studies. This implies that a more exhaustive characterization is definitely required before landing in clinical. In addition, the pharmacokinetic and pharmacodynamic responses, as well as toxicity report, are currently missing for those compounds. Therefore, although some of them appear to be very promising, their usage in medical practice does not seem to be so real. Additionally, CREB inhibitors have shown many limitations, including the reduced bioactivity in living systems and the off-targets binding. In our view, rather than chemical and structural deficiencies, off-targets could represent the result of both multifunctional domains contained within CBP and high versatility of CRE sequence to bond several partners. While the loss of target specificity constitutes a restraining factor for clinical approval, in the event of safety outline and full targets profiling, it could be a facilitating feature for therapeutic usage. The pharmacological approach is moving forward multitarget drugs in cancer treatment; for this reason, developing a synthetic compound capable of inhibiting CREB and other tumor-related factors might be useful to fight cancer. In this connection, the histone lysine demethylases JMJD3/UTX inhibitor GSK-J4 that we recently identified as a CREB modulator in AML cells might conceive a valid choice [16]. We are aware that our findings partly clarify the molecular mechanisms by which GSK-J4 impacts on CREB pathway in AML cells, and thus further investigation is absolutely needed to fully understand its effective working spectrum. Nevertheless, to corroborate our results, it must be clarified that a close correlation between CREB and epigenetic modulators is coming out [163,164,165]. However, as reported for CREB inhibitors, even for GSK-J4 there have been only preclinical studies so far, and, consequently, endorsing an immediate passage in clinical is altogether improbable.

Definitively, a faster translational approach might come from the usage of the “CREB-related pathways inhibitors”. Indeed, all the considered class-related compounds have already been approved by FDA and, thus, in the case of success, it would only be a drug repurposing. However, based on the existing findings, there is no way to elucidate whether the therapeutic benefits of those drugs can be imputable to CREB modulation or just to the inhibition of their respective targets/off-targets. Therefore, designing more focused clinical trials, in which, for instance, cancer patients are grouped according to CREB expression profile and evaluated for the relative drug responses, might provide appropriate information to the many unanswered issues.

## 7. Conclusions

The present scientific statements suggest a potential therapeutic role for CREB in cancer, even though contrasting and disorienting results occur. The high recurrence degree and the adaptive survival features make cancer a very complex and unstable disease. Moreover, acquired drug resistance further contributes to complicate the clinical outcome, representing the main limiting factor for achieving cures in cancer. Therefore, increasing the number of available anticancer compounds becomes indispensable. To date, the most consistent findings suggest that CREB can potentially represent a target therapy candidate, and, even though its clinical employment does not seem so close, at least not in the immediate future, we believe that is worth keeping on this way to give hope and future to cancer patients.

## Figures and Tables

**Table 1:** Schematic summary of major existing CREB Inhibitors.

Target	Name	Efficacy (μM)	Anticancer Properties	Other Targets	Clinical Trials
CREB:CBP	Naphthol AS-E-P	6.89 ^[a]^	Primary and T-ALL cells (Jurkat and Molt4) [81] Primary and BCP-ALL cells (Nalm6 and RS4) [81] K-RAS^V12^ transformant cells [114] HER-2/neu+ cells (MCF-7) [140]	NF-κB [112] Myb [113]	N/A
	Naphthol AS-TR-P	3.70 ^[a]^	LC cells (A549, H441, H1792, H1975, H520, H2170) [117]	E2F8 [117]	
	666-15	0.073 ^[b]^	LC cells (A549) [120] BC cells (MCF-7, MDA-MB-231, MDA-MB-468) [120] PDX model of TNBC (USTC11) [122] PDAC cells (MiaPaCa2) [123] PKT mice [123]	p53, NF-κB [120]	
CREB:CRE-DNA	NSC 12155	0.6 ^[c]^	HER-2/neu+ cells [129] GBM cells (F98) [130]	C/EBPβ [128] GAG [130]	N/A
	NSC 13778	13.9 ^[c]^	N/A	C/EBPβ, VBP, AP-1 [128] TFE3 [133]	
	P6981	0.005 ^[d]^	CCS cells (SU-CCS-1) [135]	C/EBPα, C/EBPβ [135]	
	NSC 45576	11.9 ^[c]^	N/A	C/EBPβ [128]	

(BC) Breast Cancer; (BCP-ALL) Precursor B cell Acute Lymphoblastic Leukemia; (CCS) Clear Cell Sarcoma; (GBM) Glioblastoma Multiforme; (LC) Lung Cancer; (PDAC) Pancreatic Ductal Adenocarcinoma; (T-ALL) T-cell Acute Lymphoblastic Leukemia; (TNBC) Triple Negative Breast Cancer. IC_50_ on NCI-H1734 [a] and MDA-MB-231 [b] by MTT; EC_50_ by fluorescein-labeled DNA [c]; IC_50_ by DNA binding activity [d].
